# Cistanche deserticola polysaccharide induces melanogenesis in melanocytes and reduces oxidative stress via activating NRF2/HO‐1 pathway

**DOI:** 10.1111/jcmm.15038

**Published:** 2020-02-25

**Authors:** Yibo Hu, Jinhua Huang, Yixiao Li, Ling Jiang, Yujie Ouyang, Yumeng Li, Lun Yang, Xiaojiao Zhao, Lihua Huang, Hong Xiang, Jing Chen, Qinghai Zeng

**Affiliations:** ^1^ Department of Dermatology Third Xiangya Hospital Central South University Changsha China; ^2^ Department of Urology Third Xiangya Hospital Central South University Changsha China; ^3^ Medicine Experimental Center Third Xiangya Hospital Central South University Changsha China

**Keywords:** *Cistanche deserticola* polysaccharide, depigmentation disease, melanocyte, melanogenesis, NRF2, oxidative stress

## Abstract

As a main part of pigmentation disorders, skin depigmentation diseases such as vitiligo and achromic naevus are very common and get more attention now. The pathogenesis of depigmentation includes melanocyte dysfunction and loss, which are possibly caused by heredity, autoimmunity and oxidative stress. Among them, oxidative stress plays a key role; however, few clinical treatments can deal with oxidative stress. As reported, *Cistanche deserticola* polysaccharide (CDP) is an effective antioxidant; based on that, we evaluated its role in melanocyte and further revealed the mechanisms. In this study, we found that CDP could promote melanogenesis in human epidermal melanocytes (HEMs) and mouse melanoma B16F10 cells, it also induced pigmentation in zebrafish. Furthermore, CDP could activate mitogen‐activated protein kinase (MAPK) signal pathway, then up‐regulated the expression of microphthalmia‐associated transcription factor (MITF) and downstream genes *TYR, TRP1, TRP2* and *RAB27A*. Otherwise, we found that CDP could attenuate H_2_O_2_‐induced cytotoxicity and apoptosis in melanocytes. Further evidence revealed that CDP could enhance NRF2/HO‐1 antioxidant pathway and scavenge intracellular ROS. In summary, CDP can promote melanogenesis and prevent melanocytes from oxidative stress injury, suggesting that CDP helps maintain the normal status of melanocytes. Thus, CDP may be a novel drug for the treatment of depigmentation diseases.

## Graphical abstract

Cistanche deserticola polysaccharide (CDP) promotes melanogenesis in human epidermal melanocytes via activating mitogen‐activated protein kinase (MAPK) signal pathway, then up‐regulates the expression of *MITF, TYR, TRP1, TRP2,* and *RAB27A*. Otherwise, CDP can attenuate H_2_O_2_‐induced oxidative stress in melanocytes via enhancing NRF2/HO‐1 antioxidant pathway and scavenge intracellular ROS.

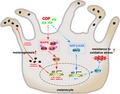

## 1 INTRODUCTION

Skin depigmentation diseases, such as vitiligo and achromic naevus, are characterized by patchy or extensive skin depigmentation.^1^ Although skin lesions seldom cause severe physical injury, they impact patient appearance and create a severe psychological burden, even mental health disorders.^2^ The major pathological changes in depigmentation include melanocyte dysfunction and loss, which greatly impacts melanin synthesis and transport,^3^ thus leading to insufficient melanin accumulation in the skin.

The mechanisms involved in depigmentation are currently unknown, but studies have identified some related factors. On the one hand, melanocyte function partly depends on microphthalmia‐associated transcription factor (MITF), which is well known for promoting the expression of melanogenesis‐related genes including tyrosinase (*TYR*), tyrosinase‐related protein 1 (*TRP1*), tyrosinase‐related protein 2 (*TRP2*), ras‐related protein Rab‐27a (*RAB27A*) and fascin actin‐bundling protein 1 (*FSCN1*).^4^ Among these genes, TYR plays a key role in melanin synthesis via oxidizing l‐dopa into dopaquinone.^5^ On the other hand, studies suggest a combination of several factors that may be responsible for melanocytes loss, including heredity, environment, autoimmunity and oxidative stress.^6^, ^7^, ^8^, ^9^ Among these factors, oxidative stress is considered the most important.

The mechanisms of oxidative stress causing depigmentation have been partly revealed; reactive oxygen species (ROS) overload is one key factor.^10^ ROS overload in depigmentation involves an imbalance between the pro‐ and antioxidant systems.^11^ One of the elements that participate in this unbalance is nuclear factor erythroid 2‐related factor 2/antioxidant response element (NRF2/ARE) antioxidant pathway impairment.^12^ The pathway consists of NRF2 and antioxidative enzymes such as haeme oxygenase‐1 (HMOX‐1, HO‐1), catalase (CAT), glutathione peroxidase 1 (GPX1) and NAD(P)H quinone dehydrogenase 1 (NQO1).^13^ When melanocytes are exposed to excessive ROS, NRF2 can translocate into the nucleus and bind to the conserved ARE, then promote the expression of antioxidant enzymes. However, in some depigmentation diseases, such as vitiligo, an impaired NRF2/ARE antioxidant pathway cannot effectively scavenge ROS.^14^

Clinical therapies commonly used in depigmentation include topical or systemic corticosteroids, calcineurin inhibitors, narrow‐band ultraviolet B (NBUVB; 311 nm), 308‐nm excimer light, autologous epidermal transplantation and traditional Chinese medicine (TCM) therapy. Corticosteroids and calcineurin inhibitors can reduce abnormal immune activation,^15^ while phototherapies are used as first‐line treatments; in particular, NBUVB stimulates melanocyte proliferation and T‐cell destruction,^16^ while 308‐nm excimer light induces T cells apoptosis.^17^ Besides, the effects of TCM therapies are related to promoting melanogenesis.^18^ To a certain extent, these methods are helpful for improving depigmentation, but controlling disease progression remains challenging. It is necessary to develop new therapies, especially for oxidative stress, which was previously neglected.

*Cistanche deserticola* is known as ‘desert ginseng’.^19^ Its components are useful in ethanol‐induced liver injury and intestinal inflammatory hyperplasia; it can also be used as an anti‐fatigue, anti‐inflammatory and anti‐tumour reagent.^20^, ^21^, ^22^ Recently, Guo et al reported that *Cistanche deserticola* polysaccharide (CDP), one of its main components, possessed antioxidant and hepatoprotective activity^20^; another two studies identified its role in protecting cells from oxidative stress injury in oxygen‐glucose deprivation/reperfusion and osteoporosis conditions.^23^, ^24^ However, the role of CDP in depigmentation diseases has not been elucidated. Here, we aimed to confirm whether CDP could affect melanogenesis and protect melanocytes from oxidative stress.

## 2 MATERIAL AND METHODS

### 2.1 Chemicals and antibodies

*Cistanche deserticola* polysaccharide (CDP) and l‐dopa were purchased from Yuanye Biotec (purity ≥ 98%; Shanghai, China). Hydrogen peroxide (H_2_O_2_), dimethyl sulphoxide (DMSO), NaOH, Triton X‐100, 4,5‐dimethylthiazol‐2‐yl‐2,5‐diphenyltetrazolium bromide (MTT) and an Annexin V‐FITC Apoptosis Detection Kit were purchased from Sigma‐Aldrich. 4% neutral paraformaldehyde was purchased from Biosharp (Hefei, China); and an Immunofluorescence Staining Kit (Alexa Fluor 488) and 2,7‐dichlorofluorescein‐diacetate (DCFH‐DA) were purchased from Beyotime Biotec (Shanghai, China). A Fontana‐Masson Stain Kit and a Nucleoplasmic Protein Extraction Kit were purchased from Sloarbio (Beijing, China). Human melanocyte growth supplement (HMGS), Dulbecco's modified Eagle medium (DMEM) and medium 254 were purchased from Gibco. Fetal bovine serum (FBS) was purchased from BI (Kibbutz Beit‐Haemek, Israel). Primary antibodies for β‐actin, TYR, TRP2, RAB27A, FSCN1, ERK, p‐ERK, JNK, p‐JNK, p38, p‐p38, NRF2 and HO‐1 were purchased from Cell Signaling Technology, primary antibody for MITF was purchased from St John's Laboratory, primary antibody for p‐MITF was purchased from Affinity Biosciences, primary antibody for GAPDH was purchased from Bioworld, and primary antibody for TRP1 was purchased from EMD Millipore.

### 2.2 Cell culture and treatment

Mouse melanoma B16F10 cells were cultured in medium DMEM supplemented with 10% FBS and 1% penicillin‐streptomycin antibiotic mix. Human epidermal melanocytes (HEMs) were separated from human foreskin (refer to our previous study^25^) and cultured in medium 254 supplemented with HMGS, 5% FBS and 1% penicillin‐streptomycin antibiotic mix. All cells were cultured in a wet incubator at 37℃ with 5% CO_2_. CDP was dissolved in DMSO and diluted with medium before use, the final concentration of DMSO was lower than 0.1%. H_2_O_2_ was diluted with medium before use.

### 2.3 Zebrafish culturing and treatment

The zebrafish embryos and medium were purchased from EzeRinka Biotech. The experimental protocol was approved by the Ethics Committee of Central South University. The zebrafish were cultured in 12‐well plates at 37°C away from light and treated with different concentrations of CDP. An inverted microscope was used to observe and record the melanins in the heads and tails of the zebrafish daily. After observation, we changed the medium and re‐added the CDP. The melanin density in the tails of zebrafish was measured with Image J, and the values are presented as integrated optical densities (IOD).

### 2.4 Cell viability

Cell viability was measured using an MTT assay. To investigate the cytotoxicity of CDP, HEMs and B16F10 cells were implanted into 96‐well plates at a density of 2 × 10^3^ cells/well and cultured until the cells attached to plates. The cells were then treated with different concentrations (0, 2.5, 5, 10, 20, 40, 80, 160 and 320 μg/mL) of CDP for 24, 48 or 72 hours. Before the measurement, 20 μL of MTT was added into each well and the plates were incubated at 37°C for 4 hours. After that, we discarded the supernatant and added 160 μL of DMSO to each well to dissolve the formazan crystals. The absorbance value at 490 nm was measured by a multimode plate reader (PerkinElmer). To investigate the effect of CDP in the H_2_O_2‐_induced cytotoxicity condition, HEMs and B16F10 cells were plated in 96‐well plates at a density of 4 × 10^3^ cells/well. The cells were treated with different concentrations of CDP (0, 20, 40 or 80 μg/mL) for 24 hours, at which point we added H_2_O_2_ (final concentrations: 500 μm for HEMs and 1.0 mm for B16F10 cells) to each well and incubated the cells for another 24 hours. We set up CDP‐treated and negative control (NC) groups. The detection steps were the same as previously described.

### 2.5 NaOH assay of melanin content

The cells were cultured in 100‐mm petri dishes and treated with CDP at different concentrations (0, 20, 40 and 80 μg/mL) for 48 hours, then digested with trypsin and collected in 1.5‐mL tubes. We washed the cells twice with double‐distilled water, resuspended them in 1 mL ethanol and vortexed them to release the melanin. We then centrifuged (200 g, 5 minutes) the mixture and discarded the supernatant, added 1 mL of 10% DMSO (diluted with 1 mm NaOH solution) into each tube and suspended the sediment. We incubated the suspension in a water bath at 80°C for 1 hour to dissolve the melanin. Finally, we transferred 200 μL of the liquid to a 96‐well plate and used a multimode plate reader to measure the absorbance value at 470 nm.

### 2.6 Tyrosinase activity measurements

The cells were cultured in 100‐mm petri dishes and treated with CDP before measurement, digested with trypsin and collected in 1.5‐mL tubes, and washed twice with phosphate‐buffered saline (PBS). We moved 10^6^ cells from each sample to a new tube and discarded the supernatant after centrifugation, then added 1 mL 0.5% Triton X‐100 to the cell pellet and stored the mixture at 0℃ for 15 minutes. Thereafter, we added 1 mL of l‐dopa (1 mm, diluted with 0.1 M phosphate buffer) as the substrate and mixed the solution, moved 200 μL of the mixture to a 96‐well plate immediately and measured the absorbance value (A0) at 475 nm using multimode plate reader, repeating the measurement at 10 minutes (A10). The tyrosinase activity was calculated by (A10‐A0)/10^5^, and the results are expressed as percentage (%) versus the negative control.

### 2.7 Fontana‐Masson melanin staining

The cells were cultured in 12‐well plates until achieving 50% density. After treatment, the cells were fixed with 4% neutral paraformaldehyde for 30 minutes and washed with distilled water. We then added 500 μL Fontana ammonia‐silver solution to each well and stored the plates in the dark for 16 hours to dye the melanin. Next, we washed the cells with distilled water for five times (1 minute each time) and soaked them in 500 μL hyposulphite for 5 minutes; finally, we removed the hyposulphite and rinsed the cells again with distilled water for 1 minute. We then used an inverted microscope to observe and record the melanins.

### 2.8 RNA extraction and quantitative reverse transcription polymerase chain reaction

The cells were cultured in 6‐well plates. After treatment, the cells were digested with trypsin and collected in 1.5‐mL tubes. We washed the cells twice with PBS, then added in 1 mL of lysis buffer, vortexed the mixture and placed the tubes on ice for 5 minutes to lyse the cells completely. The RNA was extracted using a Total RNA Kit (Omega Bio‐Tek) and reverse‐transcribed (RT) using ReverTra Ace qPCR RT Master Mix (TOYOBO). Quantitative reverse transcription polymerase chain reaction (PCR) was performed using KODSYBR qPCR Mix (TOYOBO). The reaction volume of the RT mixture was 20 μL, while the reaction volume of PCR mixture was 20 μL (cDNA 1‐8 μL, MIX 10 μL, primer F 1 μL, primer R 1 μL, add DEPC H_2_O to 20 μL). The experiments were performed as per the protocols. The sequences of the primers are listed in Table S1.

### 2.9 Protein extraction and Western blotting/immunofluorescence

The cells were cultured in 100‐mm petri dishes. After treatment, the cells were digested with trypsin and collected in 1.5‐mL tubes. We washed the cells twice with PBS, then added in 500 μL of RIPA lysis buffer (Thermo Fisher) supplemented with 1 mm phenylmethylsulphonyl fluoride (Thermo Fisher) and 1:100 diluted phosphatase inhibitor cocktail (Roche). The nuclear and cytoplasm protein was extracted using the Nucleoplasmic Protein Extraction Kit according to the manufacturer's protocol. We placed the tubes on ice for 30 minutes and vortexed them every 5 minutes to completely lyse the cells. We centrifuged the cells (200 g, 4°C, 15 minutes), moved the supernatant to a new tube and measured the concentration of total protein using a BCA protein assay kit (KeyGEN Biotec). We boiled the proteins with 5× loading buffer (Beyotime Biotec, China) at 100°C for 10 minutes, then stored them at −80°C. Polyacrylamide gel electrophoresis (PAGE) method was used in Western blotting, and 20 μg of protein of each group was separated and transferred to a polyvinylidene fluoride membrane. After antigen blocking, we incubated the membrane in 1:1000 diluted primary antibody for 16 hours at 4°C, then washed the membrane with PBST and incubated it in 1:10 000 diluted fluorescent secondary antibody for 1 hour at 37°C. The fluorescence intensity was detected using an Odyssey CLx Imaging System (LI‐COR). Immunofluorescence was performed using Immunofluorescence Staining Kit (Alexa Fluor 488) with a 1:100 dilution of the primary antibody.

### 2.10 Cell apoptosis measurement

The cells were cultured in 60‐mm petri dishes and treated with different concentrations (0, 20, 40 and 80 μg/mL) of CDP for 24 hours, and H_2_O_2_ was added (final concentrations: 500 μm for HEMs, 1.0 mm for B16F10 cells) to each well and incubated for another 24 hours. We also set up CDP‐treated and NC groups. After treatment, we digested the cells with EDTA free trypsin and collected them in tubes, then washed the cells twice with PBS and resuspended them in 100 μL of PBS. The cells were stained with an Annexin V‐FITC Apoptosis Detection Kit according to the protocol and detected by flow cytometry (FCM). FlowJo software was used to analyse the apoptosis rate.

### 2.11 Intracellular ROS measurement

The cells were cultured in 6‐well plates and treated with different concentrations (0, 20, 40 and 80 μg/mL) of CDP for 24 hours, to which we added H_2_O_2_ (final concentrations: 500 μm for HEMs, 1.0 mm for B16F10 cells) to each well, incubated them for another 24 hours and set up CDP‐treated and NC groups. After treatment, we washed cells twice with PBS to remove all the medium and FBS, then diluted the DCFH‐DA probe to 1:1000 with medium and added it to each well. We incubated the cells at 37°C for 30 minutes and washed them three times with serum‐free medium. We used an inverted fluorescence microscope to observe and record the fluorescence, then used ImageJ to measure the fluorescence intensity.

### 2.12 Statistics and analysis

The data in this work are presented as mean ± standard deviation (SD), and the statistical analysis was performed using GraphPad Prism (version 7.0) or SPSS (version 22.0), and Student's *t* test or one‐way analysis of variance (ANOVA) was used for multiple group comparisons. The grey value of the WB protein bands was standardized with GAPDH or β‐actin. Values of *P* < .05 were considered significant. All experiments were repeated at least three times.

## 3 RESULTS

### 3.1 CDP induced melanogenesis in HEMs and B16F10 cells

**Figure 1:**
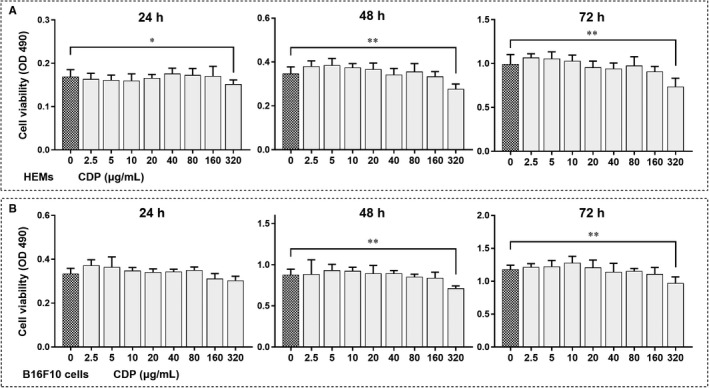
The cytotoxicity of CDP in HEMs and B16F10 cells. The cells were treated with CDP at different concentrations (0, 2.5, 5, 10, 20, 40, 80, 160 and 320 μg/mL) for 1‐3 d, and cell viability (OD value, 490 nm) was measured by MTT assay: a. the cell viability of HEMs; b. the cell viability of B16F10 cells. (*P < .05, **P < .01)

Before we started, we used the MTT assay to investigate the potential cytotoxicity of CDP on HEMs and mouse melanoma B16F10 cells. The cells were treated with CDP at different concentrations for 24, 48 or 72 hours. HEM viability testing demonstrated that when the concentrations were lower than 320 μg/mL, CDP had no influence on cell viability; however, the viability decreased significantly as the concentration reached 320 μg/mL at 24, 48 and 72 hours (*P* < .05; Figure 1A). The viability of B16F10 cells also decreased at 48 and 72 hours when CDP reached 320 μg/mL (*P* < .01) but no change was seen at lower concentrations (Figure 1B). We then preliminarily explored the role of CDP in melanogenesis and compared its effects to those of α‐melanocyte‐stimulating hormone (α‐MSH; concentrations: 20, 100, and 400 nm) and DMSO (0.1%) in HEMs. The results of the melanin staining, tyrosinase activity and melanin content assays suggested that CDP was comparable with α‐MSH for promoting melanogenesis, while 0.1% DMSO treatment made no difference (Figure S1).

**Figure 2:**
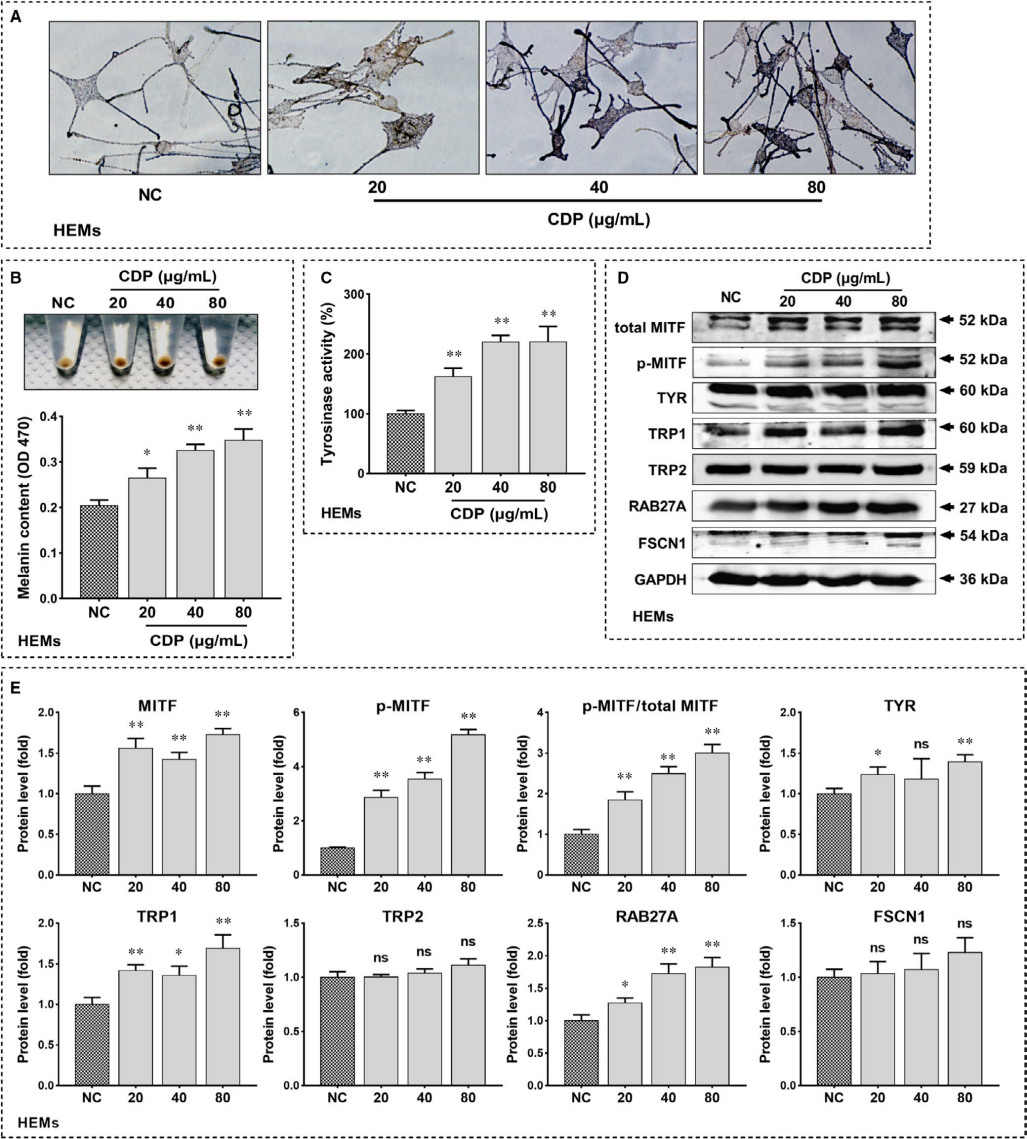
CDP promotes melanogenesis in HEMs. The HEMs were treated with CDP at different concentrations (20, 40 and 80 μg/mL) or medium alone (NC) for 48 h, then we observed the melanin by Fontana‐Masson staining, measured the melanin content (OD value, 470 nm) by NaOH assay, and measured the tyrosinase activity (OD value, 475 nm) by tyrosinase activity assay; meanwhile, we measured the protein levels of MITF, p‐MITF, TYR, TRP1, TRP2, RAB27A and FSCN1 by Western blotting and measure the grey value of protein bands by Image J: (A) melanin staining; (B) the melanin content; (C) the tyrosinase activity; (D) the protein levels of melanogenesis‐related genes; (E) the statistics of protein's grey value (standardized with GAPDH). (*P < .05, **P < .01)

We further refined our exploration accordingly. The HEMs were treated with CDP at different concentrations (20, 40 and 80 μg/mL) for 48 hours; the melanin staining, melanin content, and tyrosinase activity assays were conducted, and all showed significant increases after CDP treatment in a concentration‐dependent manner that peaked in the 80 μg/mL group (*P* < .05, Figure 2A‐C). We then measured the mRNA and protein levels of melanogenesis‐related genes (*MITF, TYR, TRP1, TRP2, RAB27A* and *FSCN1*). CDP significantly increased the mRNA levels of those genes in HEMs (*P* < .05; Figure S2A). Besides, the levels of MITF, TYR, TRP1 and RAB27A proteins increased, as did the ratio of phosphorylated MITF to total MITF (*P* < .05), whereas TRP2 and FSCN1 showed no difference (Figure 2D,E). The results indicated that CDP can promote melanogenesis and up‐regulate the expression of melanogenesis‐related genes in human melanocytes.

Furthermore, we reverified the effects of CDP again with B16F10 cells and found that the melanin content of B16F10 cells increased significantly (*P* < .01; Figure S2B). Besides, CDP significantly increased the mRNA levels of the melanogenesis‐related genes (*P* < .05; Figure S2C), and the protein levels of MITF, TYR, TRP1, TRP2 and RAB27A also increased after CDP treatment (*P* < .05; Figure S2D‐E).

### 3.2 CDP promoted melanogenesis in zebrafish

**Figure 3:**
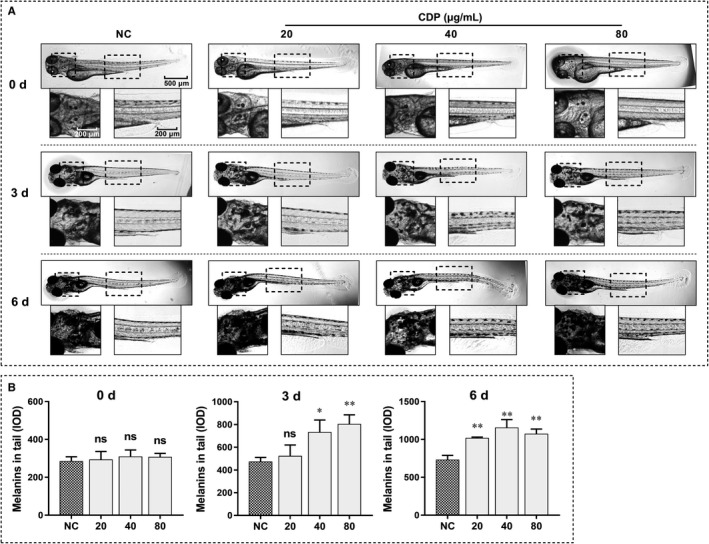
CDP promotes melanogenesis in zebrafish. The zebrafish embryos were cultured in the dark and treated with CDP at different concentrations (20, 40 and 80 μg/mL) or zebrafish medium alone (NC), then we observed the melanin granules in the heads and tails of the zebrafish daily, and took pictures using an inverted microscope; besides, we measured the melanin densities (IOD) in the tails by Image J: (A) melanins in the heads and tails of zebrafish; (B) melanin densities in zebrafish. (*P < .05, **P < .01)

To investigate whether CDP could promote melanogenesis in vivo*,* we used zebrafish embryos. The zebrafish embryos were divided into four groups and continuously treated with medium alone (NC) or CDP at different concentrations (20, 40 and 80 μg/mL); the density and distribution of melanin granules were observed and recorded every day. As the zebrafish embryos grew, we found that melanin density gradually increased in the heads and tails. On the 3rd day, the intergroup differences were distinguishable, and the difference continued to increase until we ended the experiment on the 6th day (Figure 3A). We used Image J to measure the melanin density in the tails of zebrafish; the melanin density in the CDP‐treated groups was significantly higher than that in the control group (*P* < .05; Figure 3B).

### 3.3 CDP activated MAPK signal pathway in HEMs and B16F10 cells

**Figure 4:**
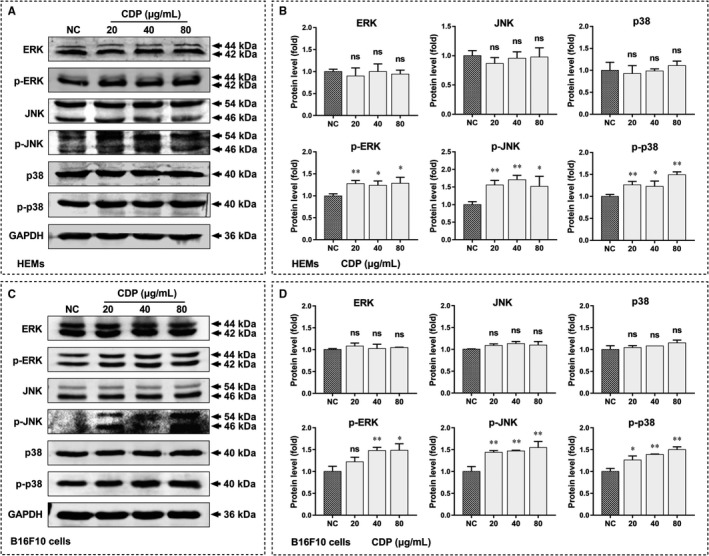
CDP activates MAPK signal pathway in HEMs and B16F10 cells. The cells were treated with CDP at different concentrations (20, 40 and 80 μg/mL) or medium alone (NC) for 48 h, then we measured the protein levels of ERK, p‐ERK, JNK, p‐JNK, p38 and p‐p38 by Western blotting and measure the grey values of protein bands by Image J: (A) the protein levels of MAPKs in HEMs; (B) the statistics of protein's grey values in HEMs (standardized with GAPDH); (C) the protein levels of MAPKs in B16F10 cells; (D) the statistics of protein's grey values in B16F10 cells (standardized with GAPDH). (*P < .05, **P < .01)

To reveal the mechanisms by which CDP promoted melanogenesis, we treated HEMs with CDP at different concentrations (20, 40 and 80 μg/mL) for 48 hours and then investigated the phosphorylated and total levels of the ERK, JNK and p38 proteins in the MAPK signalling pathway. As measured by Western blotting, the p‐ERK, p‐JNK and p‐p38 levels were increased after CDP treatment (*P* < .05), while the total levels of them did not change (Figure 4A,B). The experiments were repeated with B16F10 cells and the phosphorylated levels of the ERK, JNK and p38 proteins were increased (*P* < .05), but the total MAPK levels did not change (Figure 4C,D), consistent with the results in HEMs.

### 3.4 CDP attenuated H_2_O_2_‐induced cytotoxicity and apoptosis in HEMs and B16F10 cells

We used H_2_O_2_ to simulate the oxidative stress environment and further explored the role of CDP in melanocytes under oxidative stress. The final concentration of H_2_O_2_ used in the HEMs was 500 μm, while that in B16F10 cells was 1.0 mm. To investigate the effect of CDP on H_2_O_2_‐induced cytotoxicity, we pretreated HEMs and B16F10 cells with CDP at different concentrations (20, 40 and 80 μg/mL) for 24 hours, then added H_2_O_2_ and continued to treat them for 24 hours before performing the observation and MTT assay.

**Figure 5:**
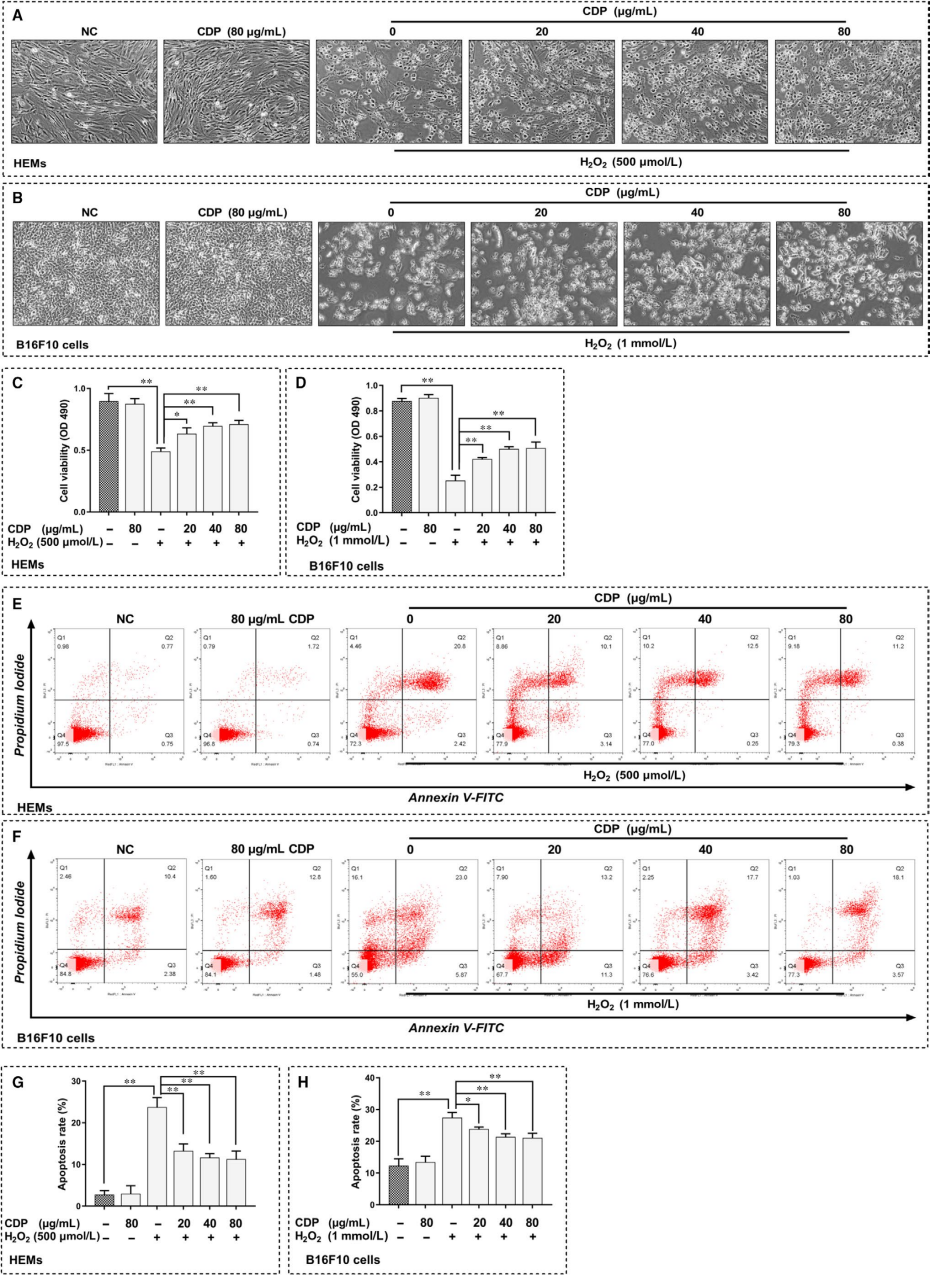
CDP attenuates H_2_O_2_‐induced cytotoxicity and apoptosis in HEMs and B16F10 cells. The cells were pretreated with CDP at different concentrations (0, 20, 40 and 80 μg/mL) for 24 h; then, the cells were treated with H_2_O_2_ (final concentrations: 500 μm for HEMs, 1.0 mm for B16F10 cells) for 24 h; besides, we set up CDP‐treated (80 μg/mL) and NC groups. After treatment, we observed the cell morphology, measured the cell viability by MTT assay, measured the apoptosis by FCM and calculated the apoptosis rate by Flow Jo: (A‐B) the cell morphology of HEMs and B16F10 cells; (C‐D). the cell viability of HEMs and B16F10 cells; (E‐F). the apoptosis of HEMs and B16F10 cells; (G‐H). the apoptosis rate of HEMs and B16F10 cells. (*P < .05, **P < .01)

H_2_O_2_ caused apparent membrane blebbing and cell shrinkage in HEMs, in the CDP‐pretreated groups, the situation was alleviated. Treatment with CDP alone had no effect (Figure 5A). The MTT assay showed a similar result in that H_2_O_2_ treatment decreased HEM viability, whereas CDP significantly ameliorated this harmful impact (*P* < .01); treatment with CDP alone made no difference (Figure 5C). We then verified the protective effect again in the B16F10 cells. H_2_O_2_ treatment induced apparent membrane blebbing and cell shrinkage, while the unfavourable condition was also ameliorated by CDP in B16F10 cells (Figure 5B). Meanwhile, the MTT assay showed that B16F10 cell viability decreased after H_2_O_2_ treatment, but the situation improved significantly in the CDP‐pretreated groups (*P* < .05; Figure 5D).

Furthermore, we used FCM to measure the H_2_O_2‐_induced apoptosis of HEMs and B16F10 cells. The results showed that H_2_O_2_ caused significant apoptosis in HEMs and B16F10 cells, but pretreatment with CDP at different concentrations could reduce the apoptosis significantly; otherwise, CDP treatment alone made no difference (Figure 5E,F). The apoptosis rate increased after H_2_O_2_ treatment but decreased in the CDP‐pretreated groups, the change was significant in both HEMs and B16F10 cells (*P* < .05, Figure 5G,H).

### 3.5 CDP scavenged H_2_O_2_‐induced intracellular ROS in HEMs and B16F10 cells

**Figure 6:**
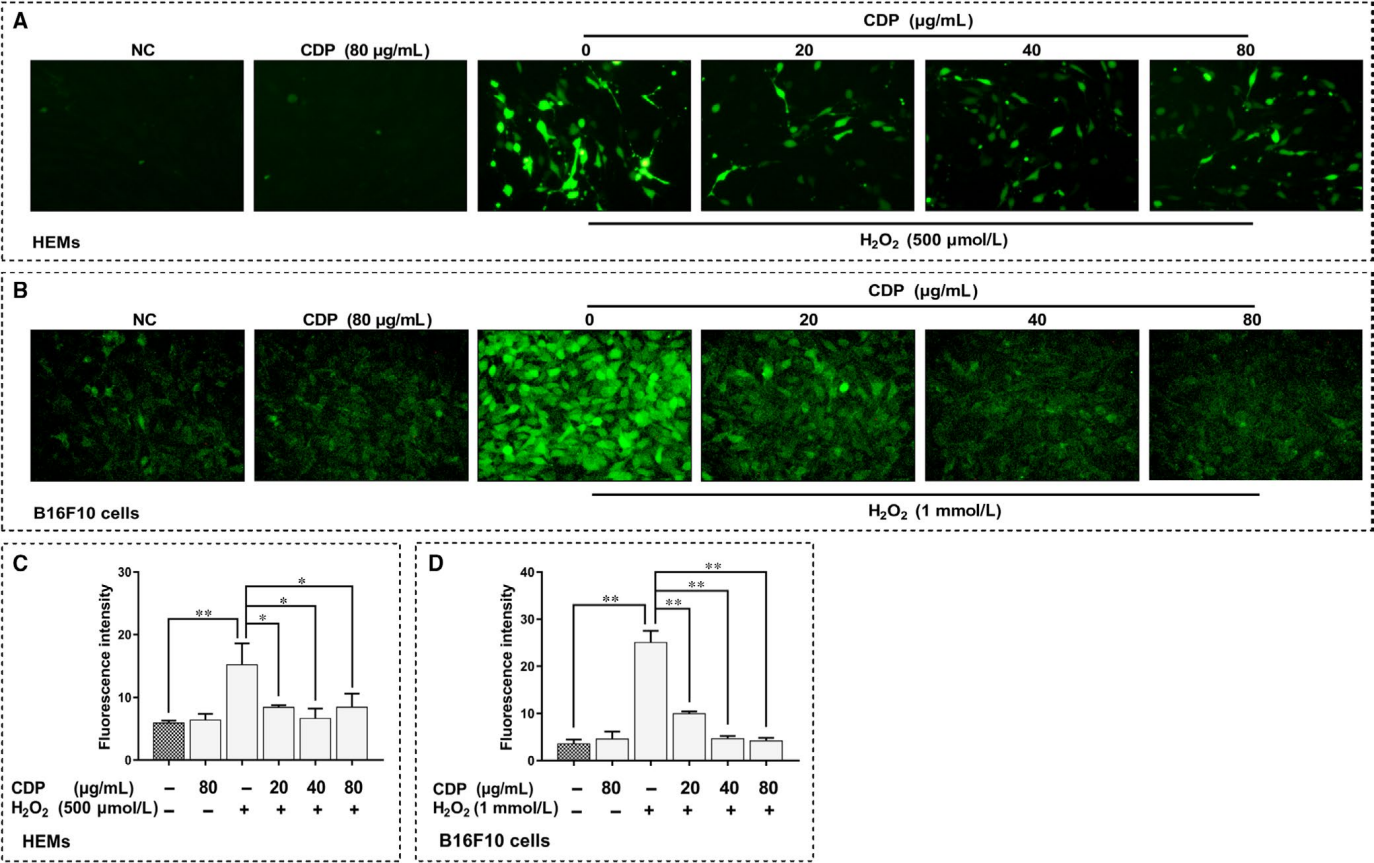
CDP scavenges H_2_O_2_‐induced intracellular ROS in HEMs and B16F10 cells. The cells were pretreated with CDP at different concentrations (0, 20, 40 and 80 μg/mL) for 24 h, then treated with H_2_O_2_ (final concentrations: 500 μm for HEMs, 1.0 mm for B16F10 cells) for 24 h; we set up CDP‐treated (80 μg/mL) and NC groups. We then stained the cells with DCFH‐DA probe, observed the fluorescence by a fluorescent microscope and measured the fluorescence intensity by Image J: (A‐B) the intracellular ROS in HEMs and B16F10 cells; (C‐D). the fluorescence intensity of ROS in HEMs and B16F10 cells (*P < .05, **P < .01)

To investigate the mechanisms of CDP for decreasing H_2_O_2_‐induced cytotoxicity and apoptosis, we further detected the intracellular ROS in HEMs and B16F10 cells using a DCFH‐DA fluorescence probe. The treatments were the same as described above, fluorescence intensity was measured to represent the ROS level. As we observed in HEMs, H_2_O_2_ treatment was effective at elevating ROS, but CDP significantly scavenged the intracellular ROS in H_2_O_2_‐treated groups (*P* < .01; Figure 6A,C); besides, the use of CDP alone made no difference. We reconfirmed the results in B16F10 cells. In B16F10 cells, treatment with CDP alone did not affect the ROS level, whereas H_2_O_2_ treatment increased the ROS level obviously, but this change was also significantly reduced by CDP pretreatment (*P* < .05; Figure 6B,D).

### 3.6 CDP up‐regulated NRF2/HO‐1 antioxidant pathway in melanocytes

**Figure 7:**
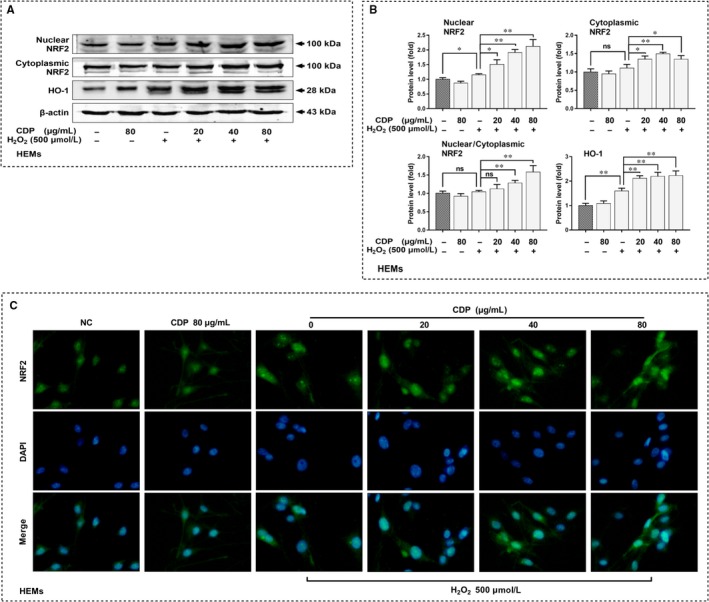
CDP up‐regulates NRF2/HO‐1 antioxidant pathway in HEMs. The cells were pretreated with CDP at different concentrations (0, 20, 40 and 80 μg/mL) for 24 h, then treated with H_2_O_2_ (final concentration: 500 μm) for 24 h; we set up CDP‐treated (80 μg/mL) and NC groups. We then measured the levels of nuclear NRF2, cytoplasmic NRF2 and total HO‐1 proteins by Western blotting, measure the grey value by Image J, and observed the NRF2 distribution by immunofluorescence: (A) the levels of NRF2 and HO‐1 proteins; (B) statistics of protein's grey value (standardized with β‐actin); (C) the distribution of NRF2. (*P < .05, **P < .01)

To further reveal the mechanisms by which CDP scavenges ROS, we firstly measured the protein levels of NRF2/HO‐1 antioxidant pathway in B16F10 cells. We found that treatment with CDP alone did not significantly affect NRF2 and HO‐1 proteins, but in H_2_O_2_‐treated groups, the level of these two proteins increased significantly, and CDP pretreatment further enhanced the elevation (*p*<.05; Figure S3). Moreover, we investigated the protein levels of NRF2 and HO‐1 in HEMs after H_2_O_2_ and/or CDP treatment. The results showed that HO‐1 level was elevated in H_2_O_2_‐treated groups, and further increased in CDP pretreatment groups (p<.05; Figure 7A, B). To know the distribution of NRF2, we compared the level of nuclear and cytoplasmic NRF2 with total level of *β*‐actin in HEMs. Similarly, we found that both nuclear and cytoplasmic NRF2 was elevated under H_2_O_2_ treatment; their levels further increased in the CDP pretreated groups. Based on that, we calculated the ratio of nuclear NRF2 to cytoplasmic NRF2. The result suggested that CDP increased the ratio significantly at the concentrations of 40 and 80 μg/mL (*p*<.05; Figure 7A, B). Using immunofluorescence, we observed the fluorescence of NRF2 in HEMs and knew that, under H_2_O_2_ treatment, the nucleus seemed to have expanded and the levels of NRF2 in the nucleus and cytoplasm were enhanced. Among them, the cells pretreated with CDP showed some differences, as we could see that the fluorescence in the nucleus was stronger than that in the cytoplasm, and the distribution was close at three CDP concentrations (Figure 7C).

## 4 DISCUSSION AND CONCLUSIONS

In this study, we investigated the role of CDP in HEMs and B16F10 cells. For the first time, we found that CDP could promote melanogenesis in melanocytes and promote pigmentation in zebrafish. A subsequent experiment showed that the MAPK signalling pathway was activated under CDP treatment. We further investigated its role in oxidative stress and found that CDP could attenuate H_2_O_2_‐induced cytotoxicity and apoptosis in melanocytes; meanwhile, CDP could activate the NRF2/HO‐1 antioxidant pathway and scavenge intracellular ROS under oxidative stress condition.

Mitogen‐activated protein kinase is a vital pathway involved in the regulation of MITF, a key transcription factor that promotes the expression of melanogenesis‐related genes and subsequently affects melanin synthesis and transport.^26^, ^27^ In our study, the activation of ERK, JNK and p38 in melanocytes was increased significantly after CDP treatment; meanwhile, the expressions of MITF/p‐MITF and MITF‐driven TYR, TRP1, TRP2 and RAB27A were up‐regulated accordingly. Thus, we suggest that CDP can promote melanogenesis via activating the MAPK pathway, but how CDP activates MAPKs remains unknown. According to recent studies, toll‐like receptor 4 (TLR4) is highly expressed in melanocytes and involved in melanogenesis.^28^, ^29^ TLR4 is an important transmembrane protein that can specifically bind lipopolysaccharide (LPS)^30^; studies reported that LPS can induce melanogenesis.^29^ Interestingly, several polysaccharides extracted from plants or mushrooms reportedly activated TLRs and downstream signalling pathways such as MAPK and nuclear factor kappa beta (NF‐κB).^31^, ^32^ Therefore, we suspect that CDP can be recognized and bound by TLRs, then activates the downstream MAPK signalling pathway and promotes melanogenesis. Furthermore, nucleotide‐binding oligomerization domain‐like receptors (NLRs) are known to recognize intracellular ligands and drive the activation of MAPK and NF‐κB signalling pathways.^33^, ^34^ As reported, polysaccharides extracted from *Ganoderma lucidum* and *Astragalus* can enter cells and affect NLRs.^35^, ^36^ Thus, it is possible that CDP can enter melanocytes and regulate the MAPK signalling pathway via NLRs. However, further studies are needed to verify this hypothesis.

Some studies to date reported the application of herbal polysaccharide in melanogenesis to inhibit melanin production.^37^, ^38^ However, in this study, CDP promoted melanogenesis in melanocytes, and this effect was further confirmed after comparison with α‐MSH. CDP is also a kind of polysaccharide extracted from herbs, we suspect that the opposite effect of CDP may be related to the structural differences between CDP and other polysaccharides. Polysaccharides are formed by polymerization of monosaccharides but are variable in monosaccharide type, monosaccharide composition, glycosidic bond, side‐chain structure and molecular weight^39^, ^40^; these factors are thought to determine their biological functions.^41^ Existing studies have proposed the structure of CDP and suggested that CDP structure subsequently influences its function.^42^ Thus, more proof is needed to fully understand CDP and confirm our hypothesis.

Melanogenesis is an important protective mechanism of resisting ultraviolet damage and maintaining body homeostasis^43^; at the same time, melanocytes are easily exposed to unfavourable environments such as ROS overload.^11^ It is known that ROS participate in promoting melanogenesis, one of the mechanisms is activating MAPKs.^44^ But studies also revealed that this effect exists only within a certain ROS level, while ROS overload impairs melanogenesis significantly.^45^ The relationship between ROS, MAPK and melanogenesis is variable under different conditions, thus, the balance between pro‐ and antioxidant systems is obviously important. In depigmentation diseases such as vitiligo, an imbalanced antioxidant system and uncontrollable ROS overload will damage melanocytes and decrease cell viability.^11^, ^46^ In this study, we used different concentrations of H_2_O_2_ to simulate the ROS overload in cells, and HEMs showed poorer tolerance to H_2_O_2_ than B16F10 cells. When melanocytes were subjected to H_2_O_2_ treatment, their viability and apoptosis rate got worse, but CDP pretreatment could partially reverse the trend. At the same time, ROS was scavenged. As reported, NRF2/ARE antioxidation pathway activation is a major methods of ROS scavenging in skin cells.^14^ In our experiments, the protein levels of NRF2 and HO‐1 in melanocytes were up‐regulated after H_2_O_2_ treatment without CDP; this means that H_2_O_2_‐induced oxidative stress can activate the NRF2/HO‐1 pathway, but it is insufficient for maintaining a redox balance and protecting the cell from injury. However, CDP pretreatment enhanced the NRF2/HO‐1 antioxidation pathway and restored the balance. Thus, we suggest that CDP can protect melanocytes from oxidative stress injury via activating the NRF2/HO‐1 antioxidation pathway and scavenging ROS.

We found that CDP treatment alone has no influence on ROS or NRF2/HO‐1 in melanocytes. This result suggests that CDP can affect redox balance under oxidative stress, but not normal conditions. Moreover, the NRF2/HO‐1 antioxidation pathway is reportedly regulated by the PI3K, NF‐κB and MAPK signalling pathways.^47^, ^48^ In our study, CDP was able to activate the MAPK signalling pathway. It is possible that CDP can activate the NRF2/HO‐1 pathway via up‐regulating MAPKs. As Slominski reported, melanocytes are stress sensors involved in a regulatory network, and their functions can change rapidly in response to the environment.^49^ We suggest that the role of CDP is influenced by melanocytes status, it can promote melanogenesis without affecting the antioxidant system under normal conditions but restore the redox balance under oxidative stress conditions. Therefore, the melanocyte function and survival can be maintained in two different ways by CDP. CDP helps melanocytes maintain homeostasis, which is also an important function of melanogenesis.^50^, ^51^

In conclusion, CDP can promote melanogenesis of melanocytes via activating the MAPK signalling pathway. CDP can improve melanocyte survival via activating the NRF2/HO‐1 antioxidant pathway and scavenging intracellular ROS under oxidative stress condition. Our findings are meaningful because they demonstrate that CDP can both promote melanogenesis and protect melanocytes from oxidative stress injury, which is possibly responsible for melanocyte dysfunction and loss. This study's findings indicate that CDP could be a novel drug in the treatment of depigmentation diseases.

## CONFLICT OF INTEREST

The authors confirm that there are no conflicts of interest.

## Data Availability

The data that support the findings of this study are available from the corresponding author upon reasonable request.
