# Supplementary figures and images for: Cistanche deserticola polysaccharide induces melanogenesis in melanocytes and reduces oxidative stress via activating NRF2/HO‐1 pathway
Source: J Cell Mol Med. 2020 Feb 25;24(7):4023–35. doi: 10.1111/jcmm.15038 (PMC7171403; doi:10.1111/jcmm.15038)

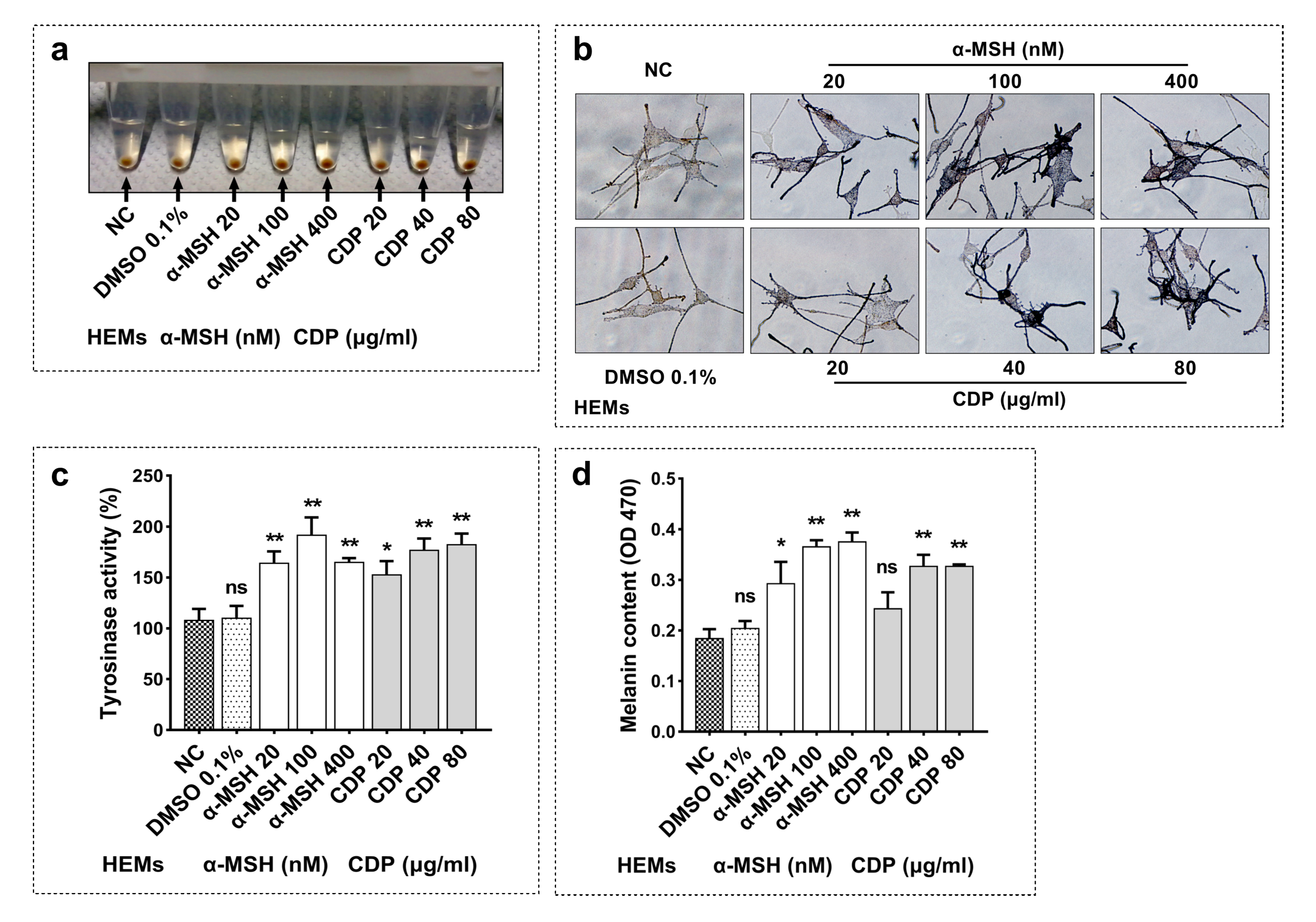

Supplement: Supplementary file 1 [file JCMM-24-4023-s001.tif]

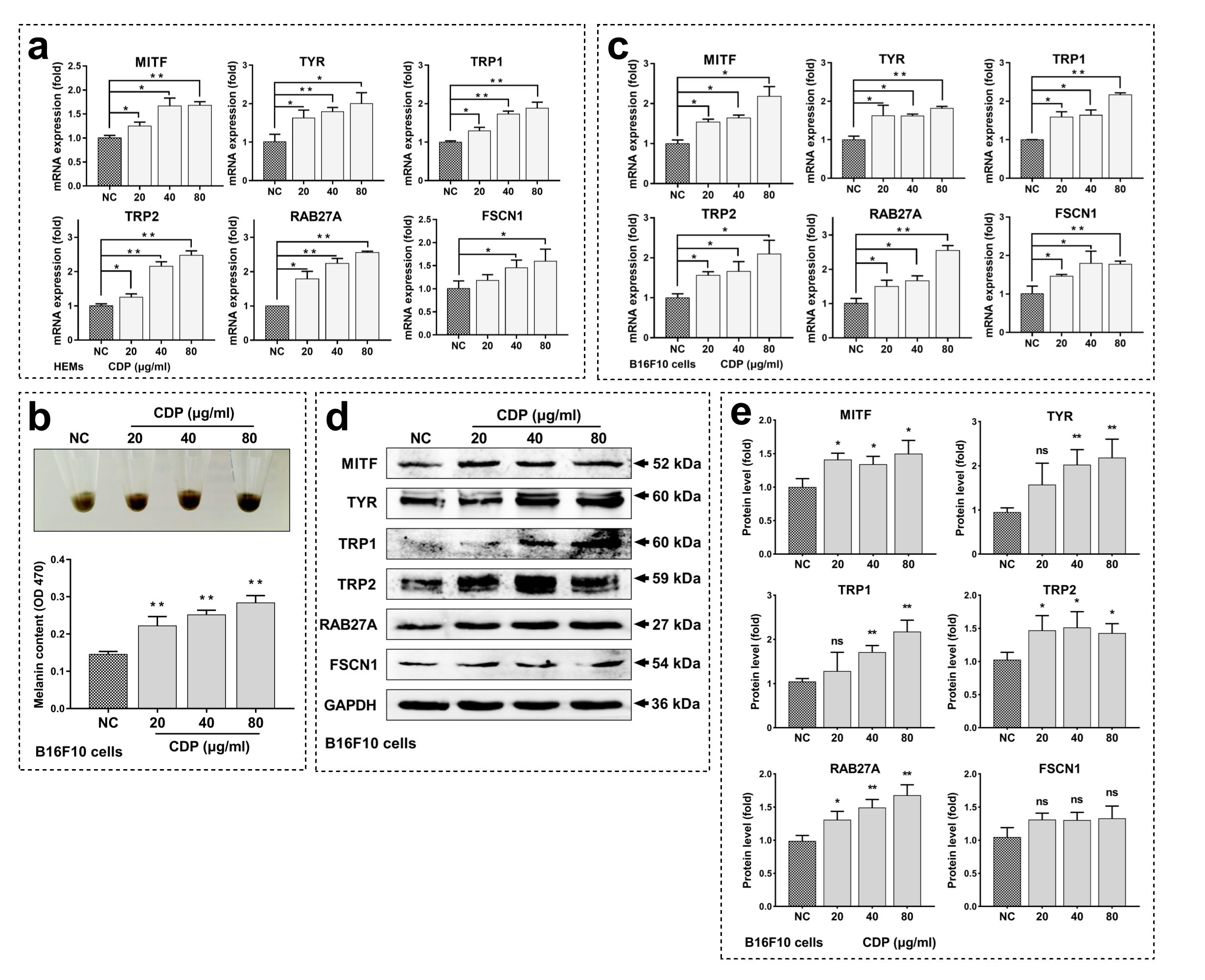

Supplement: Supplementary file 2 [file JCMM-24-4023-s002.tif]

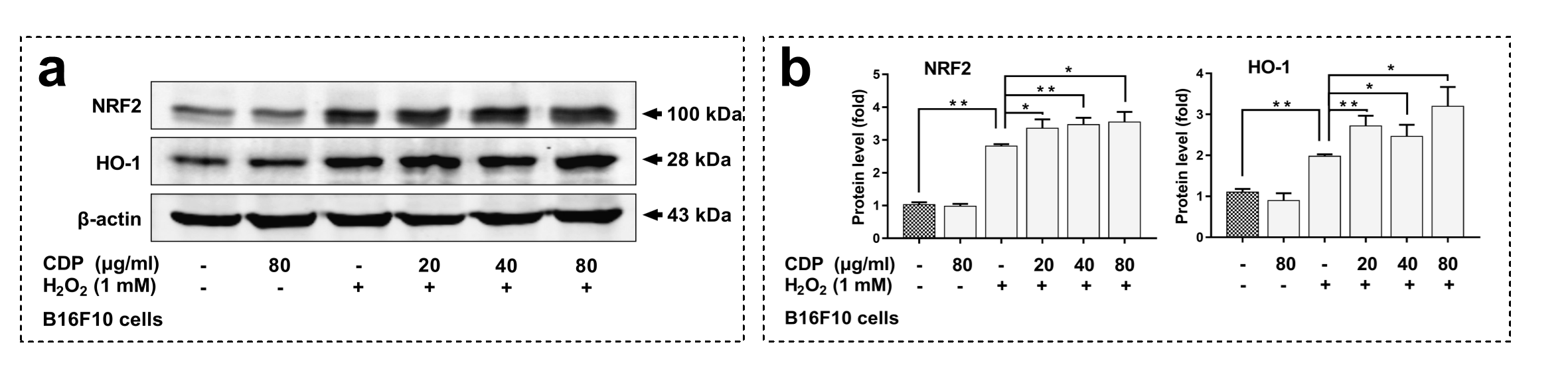

Supplement: Supplementary file 3 [file JCMM-24-4023-s003.tif]
