# Supplementary material for: Cistanche deserticola polysaccharide induces melanogenesis in melanocytes and reduces oxidative stress via activating NRF2/HO‐1 pathway
Source: J Cell Mol Med. 2020 Feb 25;24(7):4023–35. doi: 10.1111/jcmm.15038 (PMC7171403; doi:10.1111/jcmm.15038)
Supplement: Supplementary file 4 [file JCMM-24-4023-s004.docx]

**Supplement**

**Table. S1 Sequence of primers**

| **Gene symbol** | **Species** | **Forward** | **Reverse** |
| --- | --- | --- | --- |
| Gapdh | Mus | AGGTCGGTGTGAACGGATTTG | TGTAGACCATGTAGTTGAGGTCA |
| β-actin | Mus | GAGACCTTCAACACCCCAGC | ATGTCACGCACGATTTCCC |
| Mitf | Mus | AATGGCAAATACGTTACCCG | AAGGTTGGCTGGACAGGAGT |
| Tyr | Mus | AGCCCAGCATCCTTCTTCTC | AGTGGTCCCTCAGGTGTTCC |
| Trp1 | Mus | AGTGCTTGGAGGTCCGTGTA | ATTCGTCAAAGACCGCATCA |
| Trp2 | Mus | AAATAATGAGAAACTGCCAACC | CGTCTGCTTTATCAAACCCT |
| Rab27a | Mus | ACGCTATGGGTTTCCTGCTT | CCTCTTTCACTGCCCTCTGG |
| Fscn1 | Mus | ATTGGCTGCCGCAAGGTCAC | CCCGTGGAGTCTTTGATGTTGT |
| GAPDH | Homo | CTCTGCTCCTCCTGTTCGAC | GCCCAATACGACCAAATCC |
| MITF | Homo | AAATACGTTGCCTGTCTCGG | TGTTGGGAAGGTTGGCTGGA |
| TYR | Homo | TCAGCCCAGCATCATTCTTC | GGCATCCGCTATCCCAGTAA |
| TRP1 | Homo | ACCAGAGGGTTCTCATAGTCAG | TTCTCAAATTGTGGCGTGTT |
| TRP2 | Homo | GGGCAGCGAGACCAGACGAT | TTGGCAATTTCATGCTGTTTCTTC |
| RAB27A | Homo | GTAGTGAAAGAGGAGGAAGC | TCATTATCAGGTCCAGAAGC |
| FSCN1 | Homo | TGCCAATCAGGACGAGGAGAC | TCACGCCACTCGATGTCAAAGT |

**Fig. S1 CDP promotes melanogenesis in HEMs compared with α-MSH**


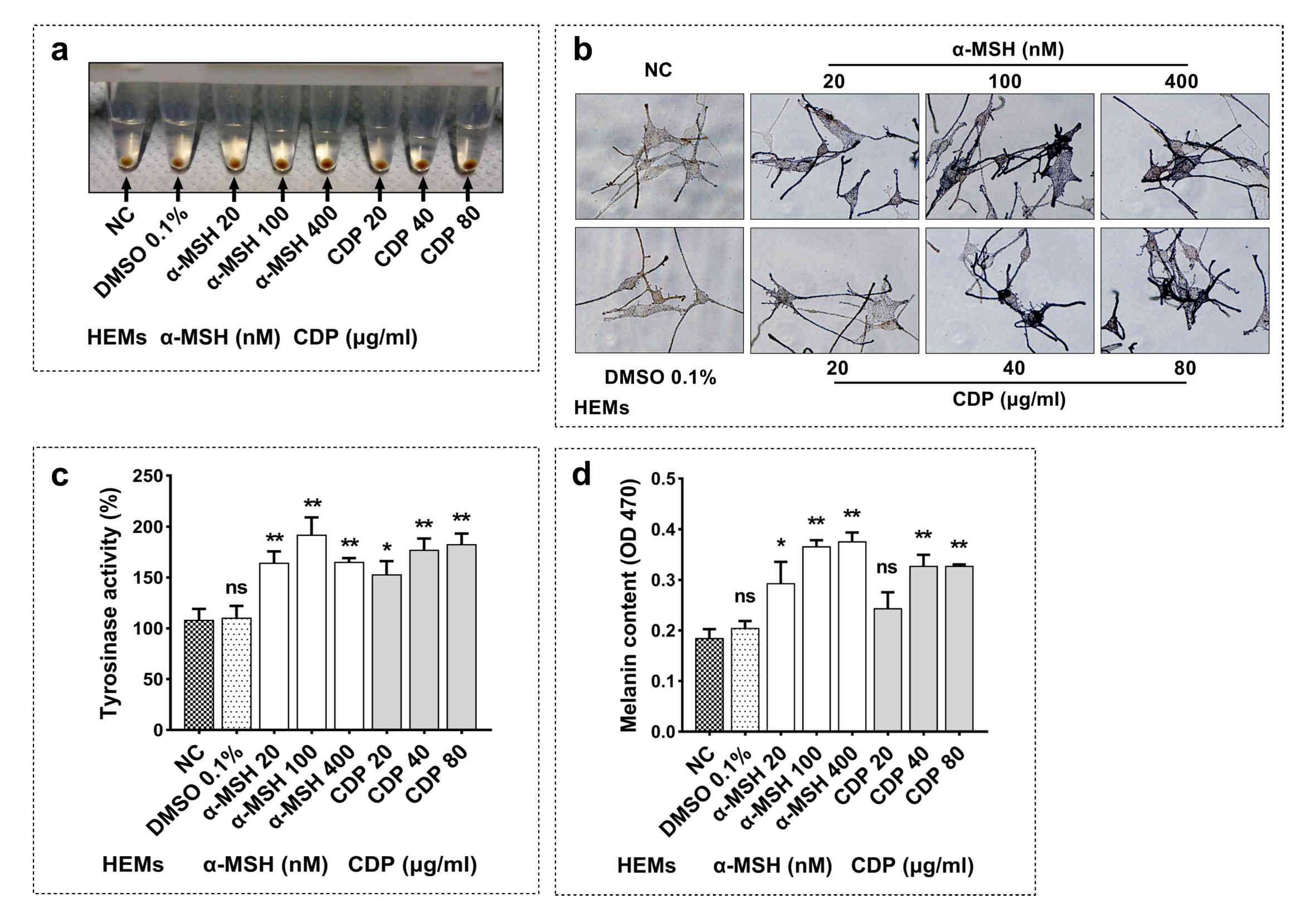


Fig. S1 CDP have comparable effects to α-MSH for promoting melanogenesis in HEMs. The HEMs were treated with α-MSH at different concentrations (20, 100, and 400 nM) or treated with CDP at different concentrations (20, 40, and 80 μg/mL), or treated with medium (NC) and DMSO (0.1%) alone for 48 h, then, we observed the melanin by Fontana-Masson staining, measured the tyrosinase activity (OD value, 475 nm) by tyrosinase activity measuring, and measured the melanin content (OD value, 470 nm) by NaOH assay: a. color of the cell precipitate; b. melanin staining; c. the tyrosinase activity; d. the melanin content. (*p < 0.05, **p < 0.01)

**Fig. S2 CDP promotes melanogenesis in HEMs and B16F10 cells**


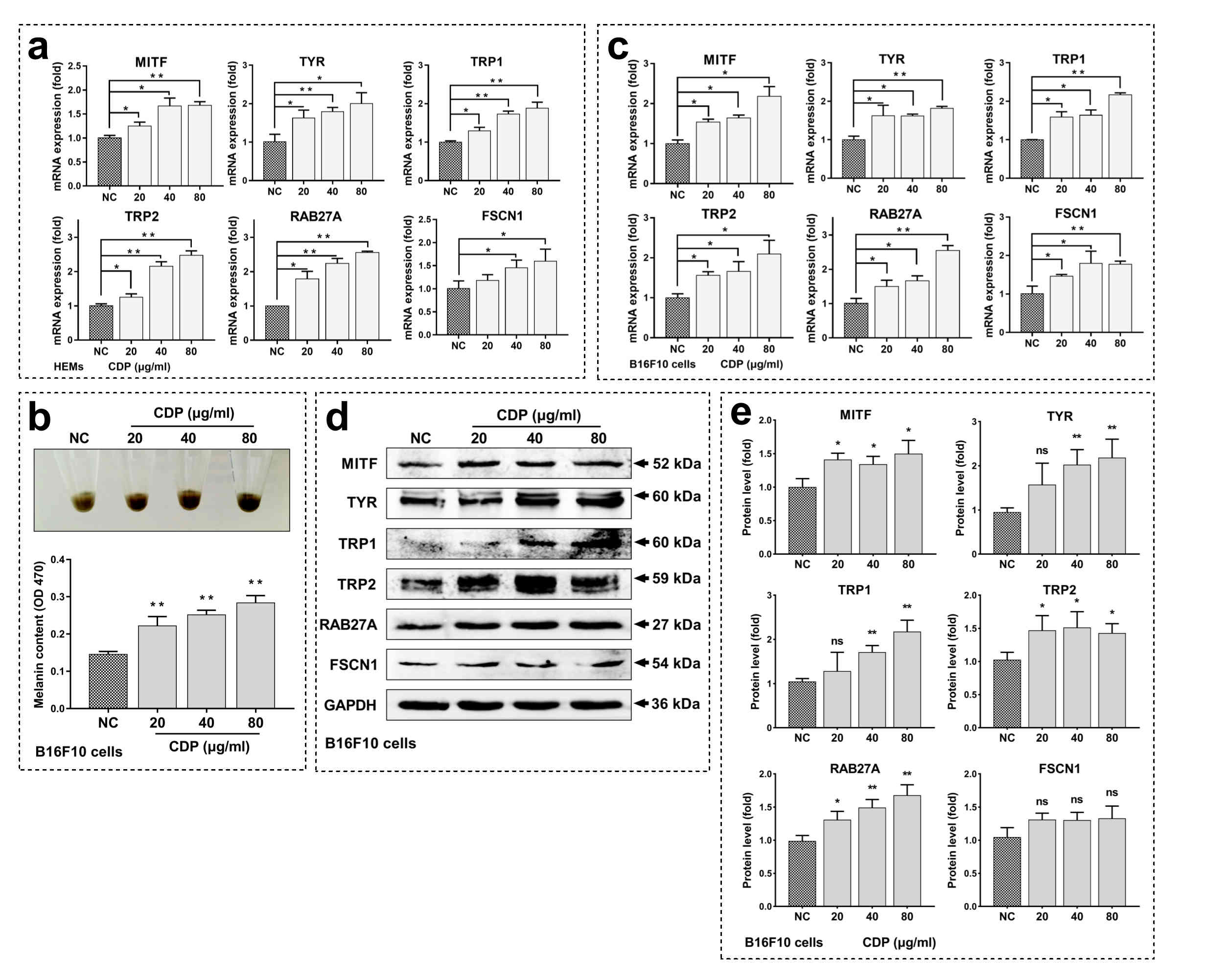


Fig. S2 CDP promotes melanogenesis in HEMs and B16F10 cells. The cells were treated with CDP at different concentrations (20, 40, and 80 μg/mL) or medium alone (NC) for 48 h; then, we measured the mRNA levels of *MITF, TYR, TRP1, TRP2, RAB27A* and *FSCN1* by qRT-PCR, measured the levels of MITF, TYR, TRP1, TRP2, RAB27A and FSCN1 proteins by western blotting: a. the mRNA levels of melanogenesis-related genes in HEMs (standardized with GAPDH); b. the melanin content in B16F10 cells; c. the mRNA levels of melanogenesis-related genes in B16F10 cells (standardized with Gapdh); d. the levels of melanogenesis-related proteins in B16F10 cells; e. statistics of protein’s gray values in B16F10 cells (standardized with GAPDH). (*p < 0.05, **p < 0.01)

**Fig. S3 CDP upregulates NRF2/HO-1 antioxidant pathway in B16F10 cells**


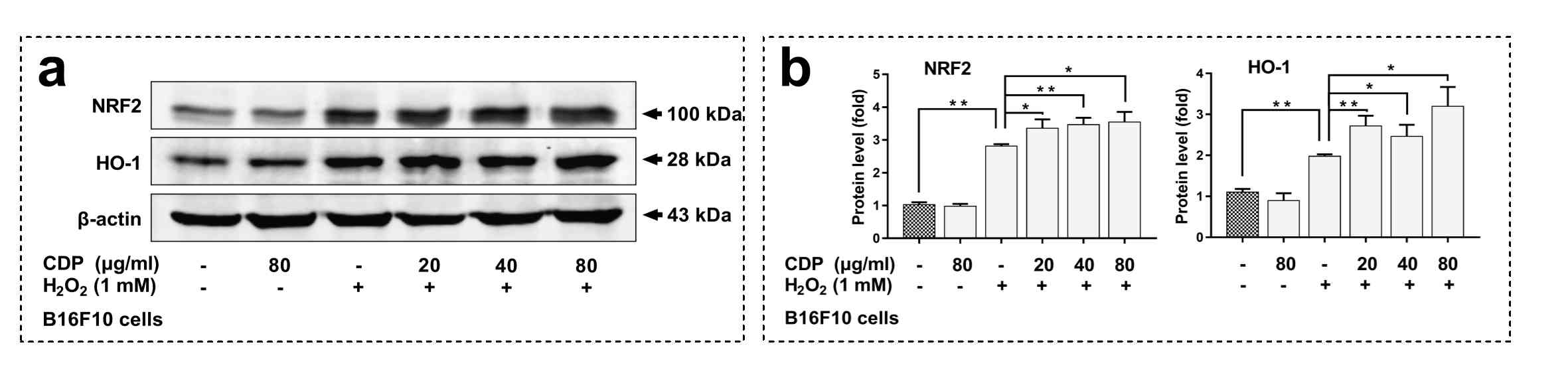


Fig. S3 CDP upregulates NRF2/HO-1 antioxidant pathway in B16F10 cells. The cells were pretreated with CDP at different concentrations (0, 20, 40, and 80 μg/mL) for 24 h, then treated with H_2_O_2_ (1 mM) for 24 h; we set up CDP-treated and negative control (NC) groups. We measured the levels of NRF2 and HO-1 proteins by western blotting and measure the gray values by Image J: a. the levels of NRF2 and HO-1 proteins; b. the statistics of protein’s gray values (standardized with β-actin). (*p < 0.05, **p < 0.01)
